# Serine Protease Inhibitor SERPINE2 Reversibly Modulates Murine Sperm Capacitation

**DOI:** 10.3390/ijms19051520

**Published:** 2018-05-19

**Authors:** Sheng-Hsiang Li, Yuh-Ming Hwu, Chung-Hao Lu, Ming-Huei Lin, Ling-Yu Yeh, Robert Kuo-Kuang Lee

**Affiliations:** 1 Department of Medical Research, Mackay Memorial Hospital, Tamsui District, New Taipei City 251, Taiwan; lsh@mmh.org.tw (S.-H.L.); hwu4416@yahoo.com.tw (Y.-M.H.); lindyyeh2008@gmail.com (L.-Y.Y.); 2 Mackay Junior College of Medicine, Nursing, and Management, Beitou District, Taipei City 112, Taiwan; huei97@yahoo.com.tw; 3 Department of Obstetrics and Gynecology, Mackay Memorial Hospital, Taipei City 104, Taiwan; d95642001@gmail.com; 4 Mackay Medical College, Sanzhi District, New Taipei City 252, Taiwan; 5 Department of Obstetrics and Gynecology, Taipei Medical University, Taipei City 110, Taiwan

**Keywords:** SERPINE2, sperm capacitation, decapacitation factor, acrosome reaction, seminal plasma

## Abstract

SERPINE2 (serpin peptidase inhibitor, clade E, member 2), predominantly expressed in the seminal vesicle, can inhibit murine sperm capacitation, suggesting its role as a sperm decapacitation factor (DF). A characteristic of DF is its ability to reverse the capacitation process. Here, we investigated whether SERPINE2 can reversibly modulate sperm capacitation. Immunocytochemical staining revealed that SERPINE2 was bound onto both capacitated and uncapacitated sperm. It reversed the increase in BSA-induced sperm protein tyrosine phosphorylation levels. The effective dose and incubation time were found to be >0.1 mg/mL and >60 min, respectively. Calcium ion levels in the capacitated sperm were reduced to a level similar to that in uncapacitated sperm after 90 min of incubation with SERPINE2. In addition, the acrosome reaction of capacitated sperm was inhibited after 90 min of incubation with SERPINE2. Oviductal sperm was readily induced to undergo the acrosome reaction using the A23187 ionophore; however, the acrosome reaction was significantly reduced after incubation with SERPINE2 for 60 and 120 min. These findings suggested that SERPINE2 prevented as well as reversed sperm capacitation in vitro. It also prevented the acrosome reaction in in vivo-capacitated sperm isolated from the oviduct. Thus, SERPINE2 could reversibly modulate murine sperm capacitation.

## 1. Introduction

Capacitation, a physiological process in the sperm, was first described in the early 1950s [1,2], in which the sperm resides in the female reproductive tract until it gains the ability to fertilize an egg. It is a complex change and is believed to be initiated by the removal of cholesterol from the sperm plasma membrane [3,4,5,6], leading to changes in the membrane structure and fluidity and increase in the permeability of the sperm for calcium and bicarbonate ions. Subsequently, a series of downstream signaling occurs, including an increase in the levels of sperm intracellular calcium ion ([Ca^2+^]_i_) and pH, thereby activating adenylyl cyclase and leading to an increase in the intracellular levels of cyclic AMP (cAMP). The activation of cAMP-dependent protein kinase then induces tyrosine phosphorylation of a subset of sperm proteins [7].

The seminal plasma is a mixture of secretions from the male accessory sexual glands, mainly containing seminal vesicle secretions. It has been shown to reverse sperm capacitation [8]. Decapacitation factors (DFs) present in the seminal plasma can reverse sperm capacitation, i.e., change sperm from a capacitated to an uncapacitated status. These factors are removed from the sperm surface before or during the capacitation process [9]. Several potential DFs have been identified in mice [10,11,12,13,14], including SERPINE2 (serpin peptidase inhibitor, clade E, member 2) [13].

SERPINE2, also known as glia-derived nexin or protease nexin-1, has broad antiprotease activity specific to serine proteases; e.g., trypsin, thrombin, plasmin, and plasminogen activator [15]. SERPINE2 is widely expressed in various tissues, including the reproductive tissues [16,17,18,19,20], with the highest levels found in seminal vesicles [13,20]. It can be purified from the seminal vesicle secretion [13].

Recent studies have demonstrated the ability of SERPINE2 to inhibit sperm capacitation by blocking the cholesterol efflux from sperm plasma membranes and suppressing the increase in the level of sperm protein tyrosine phosphorylation. These results suggested that SERPINE2 is a potential sperm DF [13]. DF has been reported to have the ability of reversing the sperm capacitation process [8]. In the present study, we investigate whether SERPINE2 can reversibly modulate sperm capacitation.

## 2. Results

### 2.1. SERPINE2 Binds to Both Capacitated and Uncapacitated Sperm

**Figure 1:**
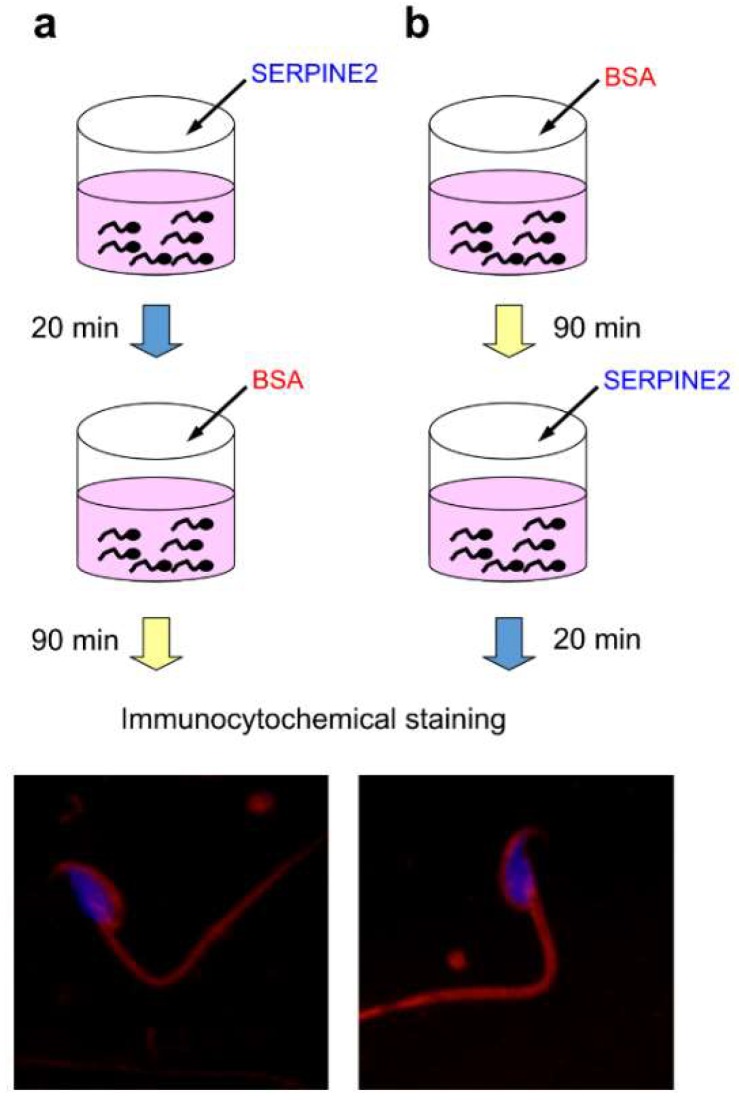
Binding of SERPINE2 on the sperm. Live epididymal spermatozoa were first cultured with 0.2 mg/mL SERPINE2 for 20 min and then incubated with 3 mg/mL BSA for an additional 90 min (**a**) or vice versa (**b**). After washing by centrifugation, spermatozoa were transferred, air-dried, and fixed onto slides. Slides were incubated with anti-SERPINE2 antiserum, treated with tetramethylrhodamine isothiocyanate (TRITC)-conjugated goat anti-rabbit IgG, and counterstained with Hoechst 33258 to localize the nuclei for contrast.

Incubation with bovine serum albumin (BSA) can induce capacitation in epididymal sperm [21]. In the immunocytochemical staining analyses, SERPINE2 was shown to bind to the acrosome, mid-piece, and principal tail of the sperm. This binding was detected regardless of whether the samples were incubated with SERPINE2 before (Figure 1a) or after (Figure 1b) the BSA-induced capacitation. These results indicated that SERPINE2 can bind to both capacitated and uncapacitated murine sperm.

### 2.2. SERPINE2 Reverses BSA-Induced Protein Tyrosine Phosphorylation in Sperm

**Figure 2:**
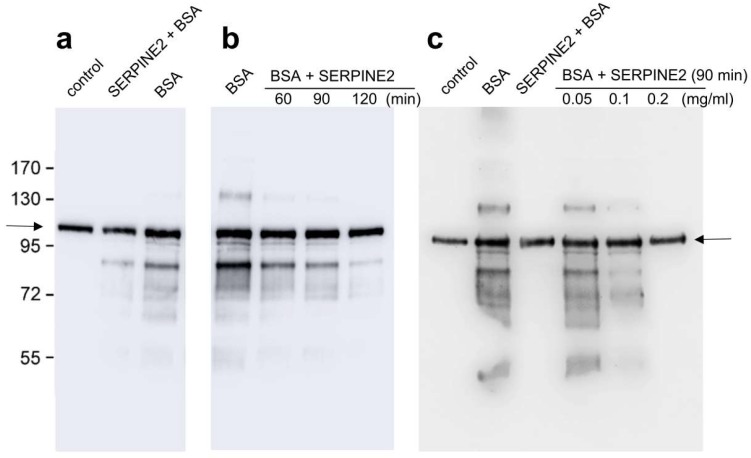
Reversible modulation of sperm protein tyrosine phosphorylation by SERPINE2. (**a**) Epididymal spermatozoa were cultured in mKRB medium in the absence (control samples) or presence of 3 mg/mL BSA at 37 °C for 90 min to allow the sperm to undergo capacitation. The sperm were first incubated with SERPINE2 (0.2 mg/mL) for 20 min prior to the addition of 3 mg/mL BSA (SERPINE2 + BSA) or (**b**) first incubated with BSA for 90 min and then treated with SERPINE2 for an additional 60, 90, and 120 min (BSA + SERPINE2); (**c**) Same as (**a**,**b**); after 90 min of BSA incubation, the sperm were treated with various dosages of SERPINE2 for 90 min. To reveal sperm protein tyrosine phosphorylation levels, the sperm extract was separated by SDS-PAGE and electrotransferred to a nitrocellulose membrane. Western blotting was performed using an anti-phosphotyrosine antibody. The arrow indicates constitutively tyrosine phosphorylated sperm hexokinase as the internal loading control.

To assess whether SERPINE2 can reverse sperm capacitation, we first established the characteristic sperm protein tyrosine phosphorylation pattern for capacitated sperm. As shown in Figure 2, murine sperm cultured in modified Krebs-Ringer bicarbonate (mKRB) medium without BSA showed a basal level of sperm protein tyrosine phosphorylation (Figure 2a, lane 1). When cultured in medium supplemented with BSA, the sperm protein tyrosine phosphorylation level was significantly increased (lane 3). However, tyrosine phosphorylation was inhibited when sperm was incubated with SERPINE2 for 20 min before BSA supplementation (lane 2). To evaluate the effective time for SERPINE2 to reverse sperm from capacitated to uncapacitated state, the sperm were incubated with SERPINE2 for 60, 90, and 120 min after BSA-induced capacitation. Levels of tyrosine phosphorylation were significantly reduced with SERPINE2 incubation times of ≥60 min (Figure 2b).

To determine the effective dosage of SERPINE2 required to reverse sperm capacitation, sperm that had been capacitated by BSA for 90 min were incubated with SERPINE2. As shown in Figure 2c, SERPINE2 dosages of ≥0.1 mg/mL effectively inhibited BSA-induced tyrosine phosphorylation.

### 2.3. SERPINE2 Reversibly Modulates Sperm Capacitation

**Figure 3:**
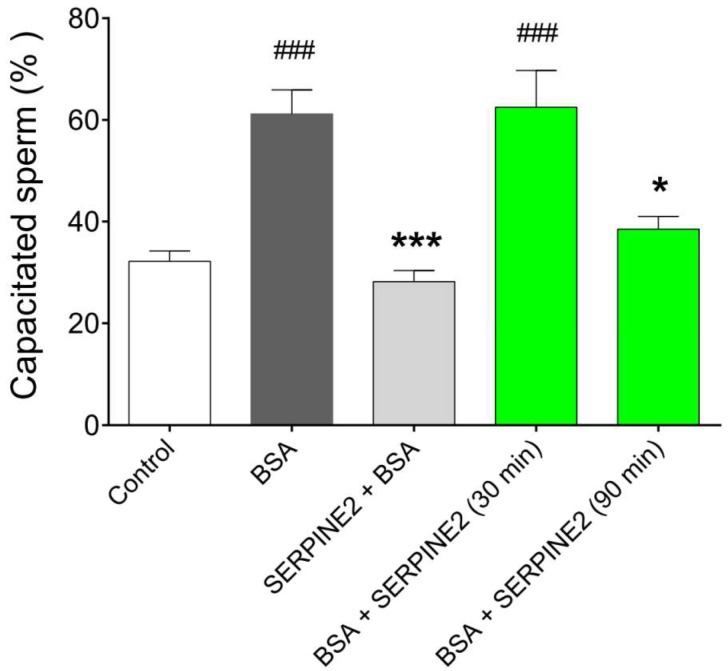
Reversible inhibition of sperm capacitation by SERPINE2 as evaluated using CTC fluorescence staining. Epididymal spermatozoa were cultured in Biggers, Whitten, and Whittingham (BWW) medium in the absence (control) or presence of BSA (3 mg/mL) for 90 min at 37 °C to allow sperm to undergo capacitation. The samples were either incubated with SERPINE2 (0.2 mg/mL) for 20 min prior to addition of 3 mg/mL BSA (SERPINE2 + BSA) or incubated with SERPINE2 for an additional 30 and 90 min after 90 min of initial BSA incubation (BSA + SERPINE2). Sperm capacitation status was evaluated using CTC fluorescence staining. ^###^ *p* < 0.001 vs. control; * *p* < 0.05, *** *p* < 0.001 vs. BSA.

To further evaluate the ability of SERPINE2 to reversibly modulate sperm capacitation, a morphological evaluation of capacitation using chlortetracycline (CTC) fluorescence staining was performed. As shown in Figure 3, control samples (incubated in medium only) exhibited the lowest percentages of capacitated sperm and were used as the baseline. BSA-treated samples had the highest percentages of capacitated sperm. Compared with BSA-treated samples, sperm capacitation was significantly inhibited in those treated with SERPINE2 before BSA supplementation. Once sperm had been capacitated by BSA, incubation with SERPINE2 for 30 min did not result in a significant change in the percentages of capacitated sperm. However, the percentages of capacitated sperm were significantly reduced in samples incubated with SERPINE2 for 90 min, indicating that SERPINE2 can reverse BSA-induced sperm capacitation.

**Figure 4:**
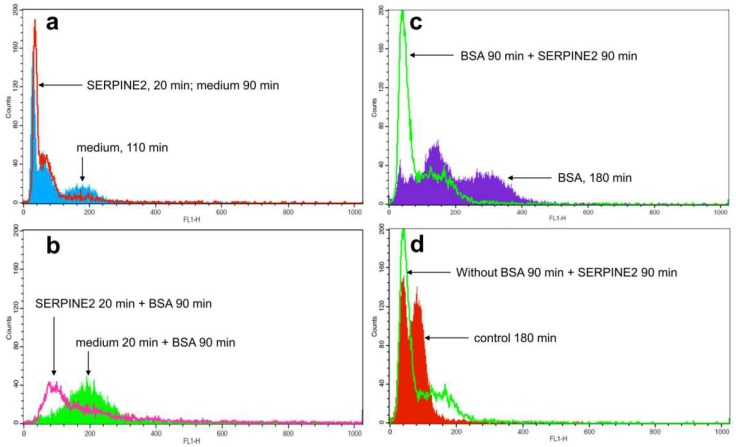
SERPINE2 reversibly inhibited the elevation of [Ca^2+^]_i_ levels induced by BSA. To detect sperm [Ca^2+^]_i_ levels, epididymal spermatozoa were preloaded with Fluo-3 AM. After washing, spermatozoa were either (**a**) cultured in BWW medium in the absence (control) or presence of SERPINE2 (0.2 mg/mL) at 37 °C for 20 min; (**b**) cultured with SERPINE2 (0.2 mg/mL) for 20 min first and then incubated with BSA (3 mg/mL) for an additional 90 min to allow the sperm to undergo capacitation, or incubated with (**c**) or without (**d**) BSA (3 mg/mL) for 90 min, followed by incubation with SERPINE2 (0.2 mg/mL) for an additional 90 min. [Ca^2+^]_i_ levels were measured by flow cytometry. The histogram shows the fluorescence intensity of sperm cells.

Ca^2+^ influx is an early event in the physiological process of murine sperm capacitation [22]. Compared with control samples, BSA-treated ones showed an increase in sperm [Ca^2+^]_i_ levels (Figure 4a,b). Samples incubated in medium supplemented only with SERPINE2 showed lower levels of [Ca^2+^]_i_ (Figure 4a); however, samples that were preincubated with SERPINE2 and then treated with BSA had significantly reduced sperm [Ca^2+^]_i_ levels (Figure 4b). To investigate whether SERPINE2 can reverse [Ca^2+^]_i_ levels in BSA-capacitated sperm, SERPINE2 was added after the sperm had been capacitated by BSA for 90 min. In these samples, sperm [Ca^2+^]_i_ levels were substantially reduced (Figure 4c). Sperm [Ca^2+^]_i_ levels were also reduced in samples that were incubated with SERPINE2 in medium without BSA (Figure 4d).

**Figure 5:**
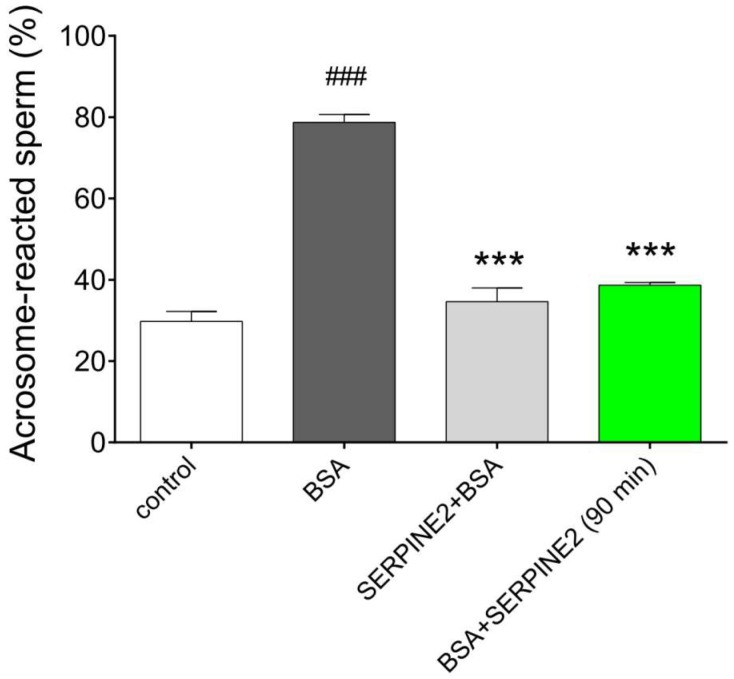
SERPINE2 reversibly inhibited the acrosome reaction in sperm. Epididymal spermatozoa were cultured in BWW medium in the absence (control) or presence of BSA (3 mg/mL) for 90 min at 37 °C to allow the sperm to undergo capacitation. Sperm samples were either incubated with SERPINE2 (0.2 mg/mL) for 20 min prior to addition of 3 mg/mL BSA (SERPINE2 + BSA) or incubated with sperm for 90 min after an initial 90-min incubation with BSA to induce capacitation (BSA + SERPINE2). The acrosome reaction was induced by the calcium ionophore A23187. Sperm capacitation status was evaluated using PNA fluorescence staining. ^###^ *p* < 0.001 vs. control; *** *p* < 0.001 vs. BSA, one-way ANOVA, followed by Bonferroni’s post hoc test.

If SERPINE2 can reversibly modulate sperm capacitation, the acrosome reaction of the capacitated sperm must be inhibited. Compared with the control samples, BSA-capacitated samples showed higher percentages of acrosome-reacted sperm (Figure 5). Preincubation with SERPINE2 inhibited BSA-induced sperm capacitation and the subsequent acrosome reaction. In samples where spermatozoa were first capacitated by BSA and then incubated with SERPINE2 for an additional 90 min, the percentage of acrosome-reacted sperm was significantly reduced.

### 2.4. SERPINE2 Suppresses the Acrosome Reaction of Sperm Isolated from the Oviduct

**Figure 6:**
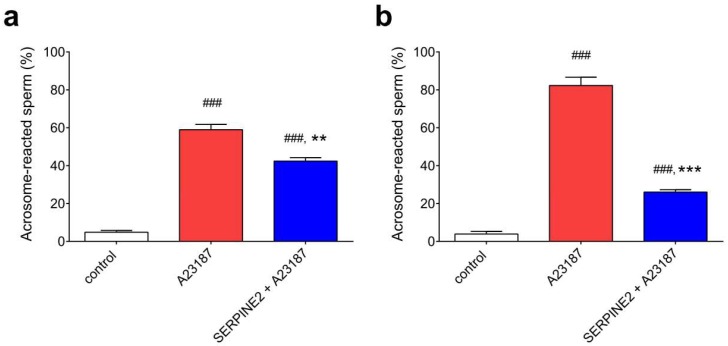
SERPINE2 inhibited oviductal sperm capacitation. Freshly flushed oviductal sperm were preincubated with 0.2 mg/mL of SERPINE2 for 60 min (**a**, *n* = 5) or 120 min (**b**, *n* = 7) or without SERPINE2 and then treated with or without A23187 (control) at 37 °C for 30 min. The sperm were smeared on a slide and fixed with methanol for 30 s. The sperm acrosomal status was assessed using TRITC-conjugated PNA staining. ^###^ *p* < 0.001 vs. control; ** *p* < 0.01, *** *p* < 0.001 vs. A23187.

To examine whether in vivo-capacitated sperm collected from the oviduct can be reversibly modulated by SERPINE2, freshly flushed oviductal sperm were incubated with 0.2 mg/mL of SERPINE2 for 60 or 90 min, followed by addition of the A23187 ionophore to induce the acrosome reaction. Control samples of oviductal spermatozoa (without A23187 treatment) almost displayed intact acrosomes (Figure 6, white bars). However, the spermatozoa were readily induced to undergo the acrosome reaction by the addition of A23187 ionophore, with approximately 60% and 80% acrosome-reacted sperm populations after 60 and 90 min, respectively (Figure 6, red bars). In contrast, in samples incubated with SERPINE2 for 60 and 120 min and then treated with A23187, the percentage of acrosome-reacted sperm dropped to 15% and 50%, respectively (Figure 6, blue bars). These results indicated that SERPINE2 can reserve the oviductal sperm from an in vivo-capacitated form to an uncapacitated state.

## 3. Discussion

Preincubation with SERPINE2 was demonstrated to inhibit BSA-induced sperm capacitation events, including cholesterol efflux, increase in sperm protein tyrosine phosphorylation, subsequent acrosome reaction, and in vitro fertilization [13]. While SERPINE2 can prevent BSA-induced sperm capacitation, it may not meet the definition of DF. In this study, we demonstrated that SERPINE2 reversibly modulated murine sperm from the capacitated to uncapacitated state, regardless of whether the sperm was capacitated in vitro or in vivo in the oviduct, thus suggesting that SERPINE2 could be a DF.

In rabbits, the seminal plasma contains DFs that can reverse the fertilization ability of capacitated sperm [8]. The DFs can be removed by centrifugation of the seminal plasma [23]. Various components of the seminal plasma, including peptides, proteins, and lipids, were suggested as candidate DFs [24,25].

Transglutaminase crosslinks SVS2, a major protein in the mouse seminal fluid, with other seminal vesicle secretion proteins to form the copulatory plug that may prevent backflow of the sperm and may prevent further mating with other males [26]. SVS2 was demonstrated to protect sperm from attack by uterus-derived cytotoxic factors [27]. In addition, SVS2 has been reported to inhibit capacitation of mouse sperm by binding with the plasma membrane ganglioside GM1 to prevent cholesterol efflux from the sperm and was suggested to be a DF [28].

In our study, capacitated sperm had to incubate with SERPINE2 for approximately 60–90 min to reverse capacitation. In contrast, capacitation reversal in rabbits was achieved in 30 min with seminal plasma treatment [8] and in 45 min in mice with SVS2 treatment [28]. Compared with these DFs, SERPINE2 does not seem to be an effective DF.

CTC staining using the fluorescent antibiotic chlortetracycline to monitor calcium-regulated changes on spermatozoa is a widely used method to examine the sperm capacitation status [29,30]. In this study, we used CTC to evaluate the effect of SERPINE2 on reversal capacitation. Although it can analyze acrosome-reacted sperm, the acrosome reaction occurs spontaneously and is different from the physiological acrosome reaction. Sperm must first be capacitated and then be induced to undergo the physiological acrosome reaction [9]. The acrosome reaction is a functional assay of capacitation; thus, when assessing SERPINE2’s impact on capacitation, we can measure the acrosome reaction to reflect the capacitation status.

The effects of genital tract secretory substances such as proteins or hormones (estrogen and progesterone) on spermatozoa can be analyzed using CTC in combination with H33258 to characterize their capacitation status [31]. The methods of assessing sperm capacitation status have been reported to apply to evaluate the fertility of large animals. Recently, Kwon et al. used CTC combined with H33258 staining to examine sperm capacitation status and applied it to evaluate boar fertility, showing an increased litter size in a field trial [32]. They also showed that combined H33258/CTC staining might be used to predict male fertility in various pig breeds [33].

When examining the effect of a protein on sperm capacitation, the distinct sperm protein tyrosine phosphorylation pattern is a good beginning. In this study, we applied the changes in levels of tyrosine phosphorylation to evaluate the effective dose and time range of SERPINE2 on reversal capacitation. Next, the experimental conditions were used for the subsequent CTC examination, sperm [Ca^2+^]_i_ analysis, and acrosome reaction evaluation. The results show similar trends with the tyrosine phosphorylation change, suggesting that SERPINE2 potentially is a DF.

SERPINE2 seemed potent to reduce sperm [Ca^2+^]_i_. Two plasma membrane proteins, i.e., Ca^2+^-ATPase and Na^+^-Ca^2+^ exchanger, export Ca^2+^ when sperm [Ca^2+^]_i_ is elevated [34]. Whether SERPINE2 activates the calcium pumping activity when reversing sperm capacitation deserves further investigation.

It is well-known that protein tyrosine phosphorylation not only reflects the capacitation status of sperm [21,35], but is also involved in various aspects of sperm function such as motility, capacitation, hyperactivation, the acrosome reaction, and fertilization [36]. Tyrosine phosphorylation and phosphotyrosine proteins that regulate male infertility have been categorized and suggested to be applied in the diagnosis and prognosis of male infertility in mammal [36].

Proteomics has been used to analyze changes in sperm proteins [37]. Proteomic analysis also reveals changes in sperm physiology before and after sperm cryopreservation, including survival, movement, and capacitation [38]. In comparing changes in protein post-translational modifications in BSA-capacitated and SERPINE2-reversed spermatozoa, the proteomic analysis may help clarify the relevant changes in sperm cells during capacitation.

Capacitation is unlikely to occur in the lower reproductive tract as capacitated sperm exhibit cholesterol efflux from their plasma membrane, leading to an increase in membrane permeability and increased likelihood of a spontaneous acrosome reaction. Capacitated sperm may thus lose the capability to fertilize an egg [4]. It is believed that sperm capacitation occurs in the oviduct. As spermatozoa enter the oviduct, they dock on the isthmic epithelial cells where they wait for capacitation signals and then move forward to the ampulla of the oviduct, where fertilization takes place [39]. Only capacitated sperm can be induced to undergo the acrosome reaction [9]; capacitated sperm can bind onto and then be induced by the zona pellucida to undergo the acrosome reaction [40]. A recent study used transgenic mouse spermatozoa labeled with enhanced green fluorescent protein in their acrosomes to trace the status of the acrosome reaction using fluorescence [41]. The results suggested that the acrosome reaction occurs within the cumulus cells.

Previous studies have reported several potential DFs in the seminal plasma of mice [10,11,12,13,14,42,43], rats [44], and humans [45,46,47]. These protein factors inhibit sperm capacitation in vitro when added prior to or simultaneously with capacitation inducers, such as BSA [10,11,12,13,42,43,44,47], cAMP analogs [14,43], or methyl-β-cyclodextrin [43]. These factors show effective inhibition of sperm capacitation in vitro. However, such factors may not meet the definition of a DF [8]. For example, two potential DFs glycodelin S [48] and SPINKL [43] were later reported to not exhibit DF activity because they could not reverse the capacitation of the oviductal sperm; these two capacitation inhibitors may be called sperm uncapacitation factors.

Only a small amount of seminal plasma, if present, can be detected in the oviduct [49,50]. In humans, seminal plasma is thought to be excluded from the cervix [51]. Thus, physiologically, DFs may not be present in the oviduct. Therefore, the DF in the seminal plasma may not act on the capacitated sperm but on uncapacitated sperm existing in the lower reproductive tract, thus preventing the premature capacitation of sperm and subsequent acrosome reaction. It is reasonable to propose that the decapacitation and uncapacitation factors in the seminal plasma protect the sperm from premature capacitation and thus preserve the fertilizing potential until the sperm reaches the oviduct.

Seminal plasma proteins do not enter the oviduct; however, some seminal proteins, such as PDC109 [52] and SERPINE2 [13], are carried by the sperm when entering the oviduct. SERPINE2 has been found on the surface of uncapacitated spermatozoa presented in the oviduct. However, SERPINE2 was gradually lost from the sperm surface membrane during the capacitation process. Once sperm had been capacitated, SERPINE2 was not detected on the sperm [13]. Interestingly, SERPINE2 has been detected in the extracellular matrix of cumulus cells surrounding the oocyte [19]; capacitated sperm must penetrate the cumulus cells to fertilize an egg. Cumulus SERPINE2 may not affect sperm capacitation due to the low levels at which SERPINE2 is found in the extracellular matrix of these cells [19].

In conclusion, SERPINE2 reverses BSA-induced capacitation events of epididymal sperm, including the increase in levels of sperm [Ca^2+^]_i_ and protein tyrosine phosphorylation, and the subsequent acrosome reaction in vitro. It also prevented the acrosome reaction in in vivo-capacitated sperm isolated from the oviduct. Thus, SERPINE2 could reversibly modulate murine sperm capacitation.

## 4. Materials and Methods

### 4.1. Animals and Sperm Preparation

Specific pathogen-free outbred ICR mice were purchased from BioLASCO Taiwan (Taipei, Taiwan) and housed under controlled lighting (14 h of light and 10 h of dark) at 21–22 °C with chow and tap water provided ad libitum. The care and use of experimental animals was reviewed and approved by the Institutional Animal Care and Use Committee of Mackay Memorial Hospital, Taipei, Taiwan (approval number: MMH-A-S-97043, 19 December 2009).

Male mice aged approximately 14 weeks were sacrificed, and the caudal epididymides were immediately removed. For each assay, four caudal epididymides removed from two mice were slit in 150 μL of prewarmed BWW media [53], with the composition of 91.5 mM NaCl, 4.6 mM KCl, 1.2 mM KH_2_PO_4_, 1.2 mM MgSO_4_∙7H_2_O, 5.6 mM glucose, 44 mM sodium lactate, 20 mM HEPES, 0.2 mM sodium pyruvate, 25 mM NaHCO_3_, and 1.7 mM CaCl_2_∙2H_2_O, pH 7.4, and incubated at 37 °C in 5% (*v*/*v*) CO_2_ for 20–30 min to allow motile sperm to swim upward. After that, the upper layer 75 μL medium containing highly motile sperm was collected and the concentration was determined using hemocytometer (Superior Marienfeld, Lauda-Königshofen, Germany).

When collecting sperm from the oviduct, we followed previous reports and collected the sperm from the oviduct at 3 h after mating as approximately 60–80% of sperm can be induced to undergo the acrosome reaction at this time [12,43]. Oviductal spermatozoa were collected by flushing both sides of the oviduct using 100 μL of phosphate-buffered saline (PBS) preheated to 37 °C filled in 1 mL syringe, and then used for the acrosome analyses.

### 4.2. Preparation of the SERPINE2 Protein and Its Antiserum

Mouse seminal vesicle secretion (SVS) was collected by squeezing the seminal vesicle of adult male mice (12–16 weeks old) and fractionated by liquid column chromatography. The SERPINE2 protein was purified from the SVS and anti-SERPINE2 antiserum was prepared according to the previously published paper [13].

### 4.3. Immunolocalization of SERPINE2 on Murine Sperm

Epididymal spermatozoa were transferred to Eppendorf tubes, suspended in BWW medium (~10^6^ cells/mL), and incubated at 37 °C in 5% (*v*/*v*) CO_2_ in humidified air. BSA (3 mg/mL) was added to the suspensions to induce sperm capacitation. Spermatozoa were either incubated with BSA for 90 min and then exposed to SERPINE2 for 20 min or were first incubated with SERPINE2 for 20 min and then exposed to BSA for 90 min. Following incubation, the sperm were washed with PBS by centrifugation at 200 *g* for 10 min. The sperm were then smeared on slides, fixed in 4% (*w*/*v*) paraformaldehyde, and allowed to air-dry. The slides were rinsed with PBS, incubated in a blocking solution (PBS containing 10% (*v*/*v*) normal goat serum) for 1 h at room temperature, and then incubated for 1 h with either anti-SERPINE2 antiserum [18] at 1:1000 dilution or with control antiserum at 1:100 dilution in blocking solution. After washing thrice with PBS for 5 min each to remove the excess antiserum, the slides were incubated for 40 min with TRITC-conjugated goat anti-rabbit IgG (1:500; Sigma-Aldrich, St. Louis, MO, USA). Next, the slides were again washed as previously described, counterstained with 5 µg/mL Hoechst 33258, and mounted in Prolong Gold antifade medium (Invitrogen Molecular Probes, Eugene, OR, USA) after briefly rinsing with PBS. The staining signal was visualized using an epifluorescence microscope equipped with a digital camera (Olympus DP 71, Tokyo, Japan).

### 4.4. Sperm Protein Tyrosine Phosphorylation

During the capacitation process, protein tyrosine phosphorylation levels in the sperm increase [35]. To examine whether SERPINE2 could reverse capacitation, approximately 5 × 10^6^ spermatozoa/mL were incubated in mKRB medium [54] containing 105 mM NaCl, 2 mM KCl, 0.35 mM NaH_2_PO_4_, 1 mM MgSO_4_∙7H_2_O, 5.60 mM glucose, 10 mM HEPES, 18.5 mM sucrose, 1.1 mM sodium pyruvate, 20 mM NaHCO_3_, and 1.7 mM CaCl_2_∙2H_2_O, pH 7.4, supplemented with 3 mg/mL BSA, at 37 °C in an atmosphere of 5% (*v*/*v*) CO_2_ in humidified air for 90 min. Then, SERPINE2 was added to examine its ability to reverse capacitation by reversing the protein tyrosine phosphorylation levels. To evaluate the effective time to reverse phosphorylation levels, sperm were treated with SERPINE2 for 60, 90, and 120 min after they were capacitated in mKRB medium containing BSA. To evaluate the effective dosage, samples were treated with 0.05, 0.1, and 0.2 mg/mL of SERPINE2 at the effective incubation time. For contrast, additional samples were incubated with SERPINE2 for 20 min before BSA supplementation, as this treatment inhibited the increase in sperm protein tyrosine phosphorylation levels [13].

After incubation, the soluble fraction of the sperm protein extracts was subjected to sodium dodecyl sulfate polyacrylamide gel electrophoresis (SDS-PAGE) on a 10% gel slab. Proteins on the gel were electrotransferred onto a nitrocellulose membrane. Western blot analyses were performed using an anti-phosphotyrosine antibody according to a previous method [35].

### 4.5. CTC Fluorescence Assay for Sperm Capacitation

Sperm capacitation was morphologically evaluated using the CTC fluorescence assay [29,30]. To assess the ability of SERPINE2 to reverse capacitation, freshly prepared epididymal spermatozoa (approximately 10^6^ cells/mL) were cultured in 50 µL of BWW medium. The sperm were incubated in a medium containing BSA (3 mg/mL), and SERPINE2 (0.2 mg/mL) was then added for an additional 30 or 90 min. The CTC fluorescence patterns, including the uncapacitated F form and capacitated B form, were examined using a digital camera on a fluorescence microscope (BX 40, Olympus, Tokyo, Japan). A random sample of 300 sperm per treatment group was evaluated.

### 4.6. Measurement of Sperm [Ca^2+^]_i_

The levels of [Ca^2+^]_i_ in sperm were evaluated by the fluorescence probe Fluo-3 AM (Invitrogen Molecular Probes, Eugene, OR, USA). In brief, freshly prepared epididymal spermatozoa (approximately 2 × 10^6^ cells/mL) were incubated for 20 min with 5 µM of Fluo-3 AM in calcium-free BWW medium at 37 °C in the dark under 5% (*v*/*v*) CO_2_ in humidified air. Following incubation, the sperm cells were washed twice by centrifugation at 100× *g* for 5 min each to remove free Fluo-3 AM. To assess the ability of SERPINE2 to reverse BSA-induced Ca^2+^ influx, Fluo-3 AM-loaded sperm were incubated in BWW medium with BSA (3 mg/mL) for 90 min and then with SERPINE2 (0.2 mg/mL) for additional 90 min. After washing twice with PBS by centrifugation at 200× *g* for 5 min each, the sperm [Ca^2+^]_i_ levels were analyzed using a flow cytometer (FACScan; Becton Dickinson Biosciences, Mountain View, CA, USA).

### 4.7. Evaluation of the Acrosome Reaction

The acrosome reaction is a functional assay of sperm capacitation; only capacitated sperm can undergo this reaction [9]. To evaluate the acrosome reaction, epididymal spermatozoa (2 × 10^6^ sperm/mL) were incubated in BWW medium with or without 3 mg/mL of BSA for 90 min as described above. To assess the ability of SERPINE2 to reverse BSA-induced sperm capacitation, SERPINE2 (0.2 mg/mL) was added, and samples were incubated for an additional 90 min after BSA-induced sperm capacitation. For contrast, additional sperm samples were preincubated with SERPINE2 for 20 min before BSA supplementation.

Generally, spermatozoa flushed from the oviduct readily undergo the acrosome reaction when induced by the A23187 ionophore [12,43]. To assess whether SERPINE2 can reverse the capacitation of sperm isolated from the oviduct, freshly washed oviductal spermatozoa were separated into three parts separately for control, A23187 ionophore positive control, and SERPINE2 pretreatment, respectively. Oviductal spermatozoa were incubated with or without SERPINE2 (0.2 mg/mL) at 37 °C under 5% (*v*/*v*) CO_2_ in humidified air for 60 min or 120 min. The spermatozoa were then treated with 10 µM A23187 ionophore in 0.1% (*v*/*v*) dimethyl sulfoxide at 37 °C for 30 min, while the control group was not treated with the A23187 ionophore. They were then collected by centrifugation at 200× *g* for 10 min, smeared onto slides, and allowed to air-dry.

The slides with epididymal or oviductal spermatozoa were immersed in methanol for 1 min, washed three times in PBS for 5 min each, and stained with 10 µg/mL TRITC-conjugated peanut agglutinin lectin (PNA; Sigma-Aldrich, St. Louis, MO, USA) in dark for 30 min. The slides were again washed three times in PBS for 5 min each, after which the spermatozoa were counterstained with Hoechst 33258 (5 µg/mL) for 30 s, mounted with Prolong Gold antifade medium (Invitrogen Molecular Probes, Eugene, OR, USA), and assessed with a fluorescence microscope (BX 40, Olympus, Tokyo, Japan). A random sample of 300 epididymal sperm or approximately 100–300 oviductal sperm per treatment group was evaluated.

### 4.8. Statistical Analysis

Data are presented as the mean ± SD. Differences between treatment groups were analyzed using the one-way ANOVA, followed by Bonferroni post hoc test using GraphPad software (GraphPad, San Diego, CA, USA). *p* < 0.05 was considered statistically significant.

## Abbreviations

BSA	bovine serum albumin
BWW	biggers, whitten, and whittingham media
[Ca^2+^]_i_	sperm intracellular calcium ion
CTC	chlortetracycline
DF	decapacitation factor
mKRB	modified Krebs-Ringer bicarbonate medium
PBS	phosphate-buffered saline
PNA	peanut agglutinin lectin
SDS-PAGE	sodium dodecyl sulfate polyacrylamide gel electrophoresis
SVS	seminal vesicle secretion
SERPINE2	serpin peptidase inhibitor, clade E, member 2
TRITC	tetramethylrhodamine isothiocyanate

## References

[1] Chang M.C. (1951). Fertilizing capacity of spermatozoa deposited into the fallopian tubes. Nature.

[2] Austin C.R. (1952). The capacitation of the mammalian sperm. Nature.

[3] Choi Y.H., Toyoda Y. (1998). Cyclodextrin removes cholesterol from mouse sperm and induces capacitation in a protein-free medium. Biol. Reprod..

[4] Cross N.L. (1998). Role of cholesterol in sperm capacitation. Biol. Reprod..

[5] Go K.J., Wolf D.P. (1985). Albumin-mediated changes in sperm sterol content during capacitation. Biol. Reprod..

[6] Shadan S., James P.S., Howes E.A., Jones R. (2004). Cholesterol efflux alters lipid raft stability and distribution during capacitation of boar spermatozoa. Biol. Reprod..

[7] Visconti P.E., Kopf G.S. (1998). Regulation of protein phosphorylation during sperm capacitation. Biol. Reprod..

[8] Chang M.C. (1957). A detrimental effect of seminal plasma on the fertilizing capacity of sperm. Nature.

[9] Yanagimachi R., Knobil E., Neill J.D. (1994). Mammalian fertilization. The Physiology of Reproduction.

[10] Fraser L.R., Harrison R.A., Herod J.E. (1990). Characterization of a decapacitation factor associated with epididymal mouse spermatozoa. J. Reprod. Fertil..

[11] Huang Y.H., Chu S.T., Chen Y.H. (2000). A seminal vesicle autoantigen of mouse is able to suppress sperm capacitation-related events stimulated by serum albumin. Biol. Reprod..

[12] Kawano N., Yoshida M. (2007). Semen-coagulating protein, svs2, in mouse seminal plasma controls sperm fertility. Biol. Reprod..

[13] Lu C.H., Lee R.K., Hwu Y.M., Chu S.L., Chen Y.J., Chang W.C., Lin S.P., Li S.H. (2011). Serpine2, a serine protease inhibitor extensively expressed in adult male mouse reproductive tissues, may serve as a murine sperm decapacitation factor. Biol. Reprod..

[14] Nixon B., MacIntyre D.A., Mitchell L.A., Gibbs G.M., O'Bryan M., Aitken R.J. (2006). The identification of mouse sperm-surface-associated proteins and characterization of their ability to act as decapacitation factors. Biol. Reprod..

[15] Scott R.W., Bergman B.L., Bajpai A., Hersh R.T., Rodriguez H., Jones B.N., Barreda C., Watts S., Baker J.B. (1985). Protease nexin. Properties and a modified purification procedure. J. Biol. Chem..

[16] Bedard J., Brule S., Price C.A., Silversides D.W., Lussier J.G. (2003). Serine protease inhibitor-e2 (serpine2) is differentially expressed in granulosa cells of dominant follicle in cattle. Mol. Reprod. Dev..

[17] Chern S.R., Li S.H., Lu C.H., Chen E.I. (2010). Spatiotemporal expression of the serine protease inhibitor, serpine2, in the mouse placenta and uterus during the estrous cycle, pregnancy, and lactation. Reprod. Biol. Endocrinol. RB&E.

[18] Lee R.K., Fan C.C., Hwu Y.M., Lu C.H., Lin M.H., Chen Y.J., Li S.H. (2011). Serpine2, an inhibitor of plasminogen activators, is highly expressed in the human endometrium during the secretory phase. Reprod. Biol. Endocrinol. RB&E.

[19] Lu C.H., Lee R.K., Hwu Y.M., Lin M.H., Yeh L.Y., Chen Y.J., Lin S.P., Li S.H. (2013). Involvement of the serine protease inhibitor, serpine2, and the urokinase plasminogen activator in cumulus expansion and oocyte maturation. PLoS ONE.

[20] Vassalli J.D., Huarte J., Bosco D., Sappino A.P., Sappino N., Velardi A., Wohlwend A., Erno H., Monard D., Belin D. (1993). Protease-nexin i as an androgen-dependent secretory product of the murine seminal vesicle. EMBO J..

[21] Visconti P.E., Moore G.D., Bailey J.L., Leclerc P., Connors S.A., Pan D., Olds-Clarke P., Kopf G.S. (1995). Capacitation of mouse spermatozoa. II. Protein tyrosine phosphorylation and capacitation are regulated by a camp-dependent pathway. Development.

[22] Xia J., Ren D. (2009). The bsa-induced ca2+ influx during sperm capacitation is catsper channel-dependent. Reprod. Biol. Endocrinol. RB&E.

[23] Bedford J.M., Chang M.C. (1962). Removal of decapacitation factor from seminal plasma by high-speed centrifugation. Am. J. Physiol..

[24] Chernoff H.N., Dukelow W.R. (1969). Decapacitation factor purification with lipid solvents. J. Reprod. Fertil..

[25] Oliphant G., Reynolds A.B., Thomas T.S. (1985). Sperm surface components involved in the control of the acrosome reaction. Am. J. Anat..

[26] Schneider M.R., Mangels R., Dean M.D. (2016). The molecular basis and reproductive function(s) of copulatory plugs. Mol. Reprod. Dev..

[27] Kawano N., Araki N., Yoshida K., Hibino T., Ohnami N., Makino M., Kanai S., Hasuwa H., Yoshida M., Miyado K. (2014). Seminal vesicle protein svs2 is required for sperm survival in the uterus. Proc. Natl. Acad. Sci. USA.

[28] Araki N., Trencsenyi G., Krasznai Z.T., Nizsaloczki E., Sakamoto A., Kawano N., Miyado K., Yoshida K., Yoshida M. (2015). Seminal vesicle secretion 2 acts as a protectant of sperm sterols and prevents ectopic sperm capacitation in mice. Biol. Reprod..

[29] Lee M.A., Storey B.T. (1985). Evidence for plasma membrane impermeability to small ions in acrosome-intact mouse spermatozoa bound to mouse zonae pellucidae, using an aminoacridine fluorescent ph probe: Time course of the zona-induced acrosome reaction monitored by both chlortetracycline and pH probe fluorescence. Biol. Reprod..

[30] Ward C.R., Storey B.T. (1984). Determination of the time course of capacitation in mouse spermatozoa using a chlortetracycline fluorescence assay. Dev. Biol..

[31] Ryu D.Y., Kim Y.J., Lee J.S., Rahman M.S., Kwon W.S., Yoon S.J., Pang M.G. (2014). Capacitation and acrosome reaction differences of bovine, mouse and porcine spermatozoa in responsiveness to estrogenic compounds. J. Anim. Sci. Technol..

[32] Kwon W.S., Rahman M.S., Lee J.S., You Y.A., Pang M.G. (2015). Improving litter size by boar spermatozoa: Application of combined h33258/ctc staining in field trial with artificial insemination. Andrology.

[33] Kwon W.S., Shin D.H., Ryu D.Y., Khatun A., Rahman M.S., Pang M.G. (2018). Applications of capacitation status for litter size enhancement in various pig breeds. Asian-Aust. J. Anim. Sci..

[34] Wennemuth G., Babcock D.F., Hille B. (2003). Calcium clearance mechanisms of mouse sperm. J. Gen. Physiol..

[35] Visconti P.E., Bailey J.L., Moore G.D., Pan D., Olds-Clarke P., Kopf G.S. (1995). Capacitation of mouse spermatozoa. I. Correlation between the capacitation state and protein tyrosine phosphorylation. Development.

[36] Kwon W.S., Rahman M.S., Pang M.G. (2014). Diagnosis and prognosis of male infertility in mammal: The focusing of tyrosine phosphorylation and phosphotyrosine proteins. J. Proteome Res..

[37] Oliva R., de Mateo S., Estanyol J.M. (2009). Sperm cell proteomics. Proteomics.

[38] Yoon S.J., Rahman M.S., Kwon W.S., Ryu D.Y., Park Y.J., Pang M.G. (2016). Proteomic identification of cryostress in epididymal spermatozoa. J. Anim. Sci. Biotechnol..

[39] Suarez S.S., Pacey A.A. (2006). Sperm transport in the female reproductive tract. Hum. Reprod. Update.

[40] Bleil J.D., Wassarman P.M. (1983). Sperm-egg interactions in the mouse: Sequence of events and induction of the acrosome reaction by a zona pellucida glycoprotein. Dev. Biol..

[41] Jin M., Fujiwara E., Kakiuchi Y., Okabe M., Satouh Y., Baba S.A., Chiba K., Hirohashi N. (2011). Most fertilizing mouse spermatozoa begin their acrosome reaction before contact with the zona pellucida during in vitro fertilization. Proc. Natl. Acad. Sci. USA.

[42] Lin M.H., Lee R.K., Hwu Y.M., Lu C.H., Chu S.L., Chen Y.J., Chang W.C., Li S.H. (2008). Spinkl, a kazal-type serine protease inhibitor-like protein purified from mouse seminal vesicle fluid, is able to inhibit sperm capacitation. Reproduction.

[43] Tseng H.C., Lee R.K., Hwu Y.M., Lu C.H., Lin M.H., Li S.H. (2013). Mechanisms underlying the inhibition of murine sperm capacitation by the seminal protein, spinkl. J. Cell. Biochem..

[44] Zhou Y., Zheng M., Shi Q., Zhang L., Zhen W., Chen W., Zhang Y. (2008). An epididymis-specific secretory protein hongres1 critically regulates sperm capacitation and male fertility. PLoS ONE.

[45] De Lamirande E., Yoshida K., Yoshiike T.M., Iwamoto T., Gagnon C. (2001). Semenogelin, the main protein of semen coagulum, inhibits human sperm capacitation by interfering with the superoxide anion generated during this process. J. Androl..

[46] Lee R.K., Tseng H.C., Hwu Y.M., Fan C.C., Lin M.H., Yu J.J., Yeh L.Y., Li S.H. (2018). Expression of cystatin c in the female reproductive tract and its effect on human sperm capacitation. Reprod. Biol. Endocrinol. RB&E.

[47] Martins S.G., Miranda P.V., Brandelli A. (2003). Acrosome reaction inhibitor released during in vitro sperm capacitation. Int. J. Androl..

[48] Chiu P.C., Chung M.K., Tsang H.Y., Koistinen R., Koistinen H., Seppala M., Lee K.F., Yeung W.S. (2005). Glycodelin-s in human seminal plasma reduces cholesterol efflux and inhibits capacitation of spermatozoa. J. Biol. Chem..

[49] Carballada R., Esponda P. (1997). Fate and distribution of seminal plasma proteins in the genital tract of the female rat after natural mating. J. Reprod. Fertil..

[50] Jappel D. (1992). Rapid transport of seminal immunoglobulin to distal parts of the female reproductive tract in rabbits. J. Reprod. Fertil..

[51] Brannigan R., Lipshultz L. (2008). Sperm transport and capacitation. Women’s Medicine.

[52] Suarez S.S. (2008). Regulation of sperm storage and movement in the mammalian oviduct. Int. J. Dev. Biol..

[53] Biggers J.D., Whitten W.K., Whittingham D.G., Daniels J.C. (1971). The culture of mouse embryos in vitro. Methods in Mammalian Embryology.

[54] Lee M.A., Storey B.T. (1986). Bicarbonate is essential for fertilization of mouse eggs: Mouse sperm require it to undergo the acrosome reaction. Biol. Reprod..

